# Low-intensity electromagnetic fields induce human cryptochrome to modulate intracellular reactive oxygen species

**DOI:** 10.1371/journal.pbio.2006229

**Published:** 2018-10-02

**Authors:** Rachel M. Sherrard, Natalie Morellini, Nathalie Jourdan, Mohamed El-Esawi, Louis-David Arthaut, Christine Niessner, Francois Rouyer, Andre Klarsfeld, Mohamed Doulazmi, Jacques Witczak, Alain d’Harlingue, Jean Mariani, Ian Mclure, Carlos F. Martino, Margaret Ahmad

**Affiliations:** 1 Sorbonne Université, CNRS Unit Biological Adaptation and Ageing, Team Repairing Neural Networks, Paris, France; 2 Sorbonne Université, CNRS Unit Biological Adaptation and Ageing, Photobiology Team, Paris, France; 3 Botany Department, Faculty of Science, Tanta University, Tanta, Egypt; 4 Department of Earth and Environmental Sciences, Ludwig-Maximillians-Universität Munich, Theresienstraße, Munich, Germany; 5 Institut des Neurosciences Paris-Saclay, Université Paris Sud, CNRS, Université Paris-Saclay, Gif-sur-Yvette, France; 6 Brain Plasticity Unit, UMR 8249 (ESPCI Paris/CNRS), PSL Research University, Paris, France; 7 Department of Biomedical Engineering, Florida Institute of Technology, Melbourne, Florida, United States of America; 8 Xavier University, Cincinnati, Ohio, United States of America; Research Institute of Molecular Pathology, Austria

## Abstract

Exposure to man-made electromagnetic fields (EMFs), which increasingly pollute our environment, have consequences for human health about which there is continuing ignorance and debate. Whereas there is considerable ongoing concern about their harmful effects, magnetic fields are at the same time being applied as therapeutic tools in regenerative medicine, oncology, orthopedics, and neurology. This paradox cannot be resolved until the cellular mechanisms underlying such effects are identified. Here, we show by biochemical and imaging experiments that exposure of mammalian cells to weak pulsed electromagnetic fields (PEMFs) stimulates rapid accumulation of reactive oxygen species (ROS), a potentially toxic metabolite with multiple roles in stress response and cellular ageing. Following exposure to PEMF, cell growth is slowed, and ROS-responsive genes are induced. These effects require the presence of cryptochrome, a putative magnetosensor that synthesizes ROS. We conclude that modulation of intracellular ROS via cryptochromes represents a general response to weak EMFs, which can account for either therapeutic or pathological effects depending on exposure. Clinically, our findings provide a rationale to optimize low field magnetic stimulation for novel therapeutic applications while warning against the possibility of harmful synergistic effects with environmental agents that further increase intracellular ROS.

## Author summary

Repetitive low-intensity magnetic stimulation has been used in the treatment of disease for over 50 years. Associated benefits have included alleviation of depression, memory loss, and symptoms of Parkinson disease, as well as accelerated bone and wound healing and the treatment of certain cancers, independently of surgery or drugs. However, the cellular mechanisms underlying these effects remain unclear. Here, we demonstrate that repetitive magnetic field exposure in human cells stimulates production of biological stress response chemicals known as reactive oxygen species (ROS). At moderate doses, we find that reactive oxygen actively stimulates cellular repair and stress response pathways, which might account for the observed therapeutic effects to repetitive magnetic stimulation. We further show that this response requires the function of a well-characterized, evolutionarily conserved flavoprotein receptor known as cryptochrome, which has been implicated in magnetic sensing in organisms ranging from plants to flies, including migratory birds. We conclude that exposure to weak magnetic fields induces the production of ROS in human cells and that this process requires the presence of the cryptochrome receptor.

**Editor’s Note**:

This Short Report received positive reviews by experts. The Academic Editor has written an accompanying Primer that we are publishing alongside this article (https://doi.org/10.1371/journal.pbio.3000018). The linked Primer presents a complementary expert perspective; it discusses considerations about the status of knowledge and experimental systems in the field that encourage cautious interpretation.

## Introduction

Weak electromagnetic radiation (μT-mT), which increasingly pollutes our environment, has been associated with dual and seemingly contradictory effects on human health. On the one hand, possibly deleterious public health consequences have elicited considerable debate on safety and exposure limits to electromagnetic field (EMF) radiation [1–4]. On the other hand, weak magnetic fields have been applied as therapeutic tools, notably in the form of pulsed electromagnetic fields (PEMFs), which have shown benefits in a broad range of regenerative medicine therapeutics, as well as in the alleviation of depression, reducing symptoms of Parkinson disease, and reducing memory loss [5–10]. Such PEMFs also affect nonexcitable tissues [7,9] and are below firing threshold for neurons [11,12] consistent with magnetic field effects and thereby activation of a biological magnetoreceptor. The current challenge is therefore to identify these putative magnetosensor(s) and to propose a mechanism that may explain the seemingly disparate effects of EMFs in medicine and in public health.

A possible class of biological magnetoreceptor [13] are the cryptochromes, which are conserved flavoprotein receptors [14] implicated in magnetosensing in organisms ranging from plants to migratory birds [14–16]. Cryptochrome receptors undergo redox reactions in the course of their activation that lead to the synthesis of reactive oxygen species (ROS) [17–19]. ROS are global regulators that are implicated in numerous cellular signaling functions related to response to stress and ageing and are toxic at high concentrations [20–23]. In mammalian cells, cryptochromes are both cytosolic and nuclear proteins that have been characterized for a role as core components of the circadian clock [24, 25] but that are not known to respond to external magnetic fields. However, recombinant mammalian cryptochromes expressed in a heterologous *Drosophila* system are reported to confer magnetic sensitivity in behavioral assays in flies [16], and they were recently proposed to play a role as sensors of low EMFs in the onset of childhood leukemia [26]. This raises the question of whether cryptochromes could be implicated in magnetic sensitivity in humans.

## Results

**Fig 1:**
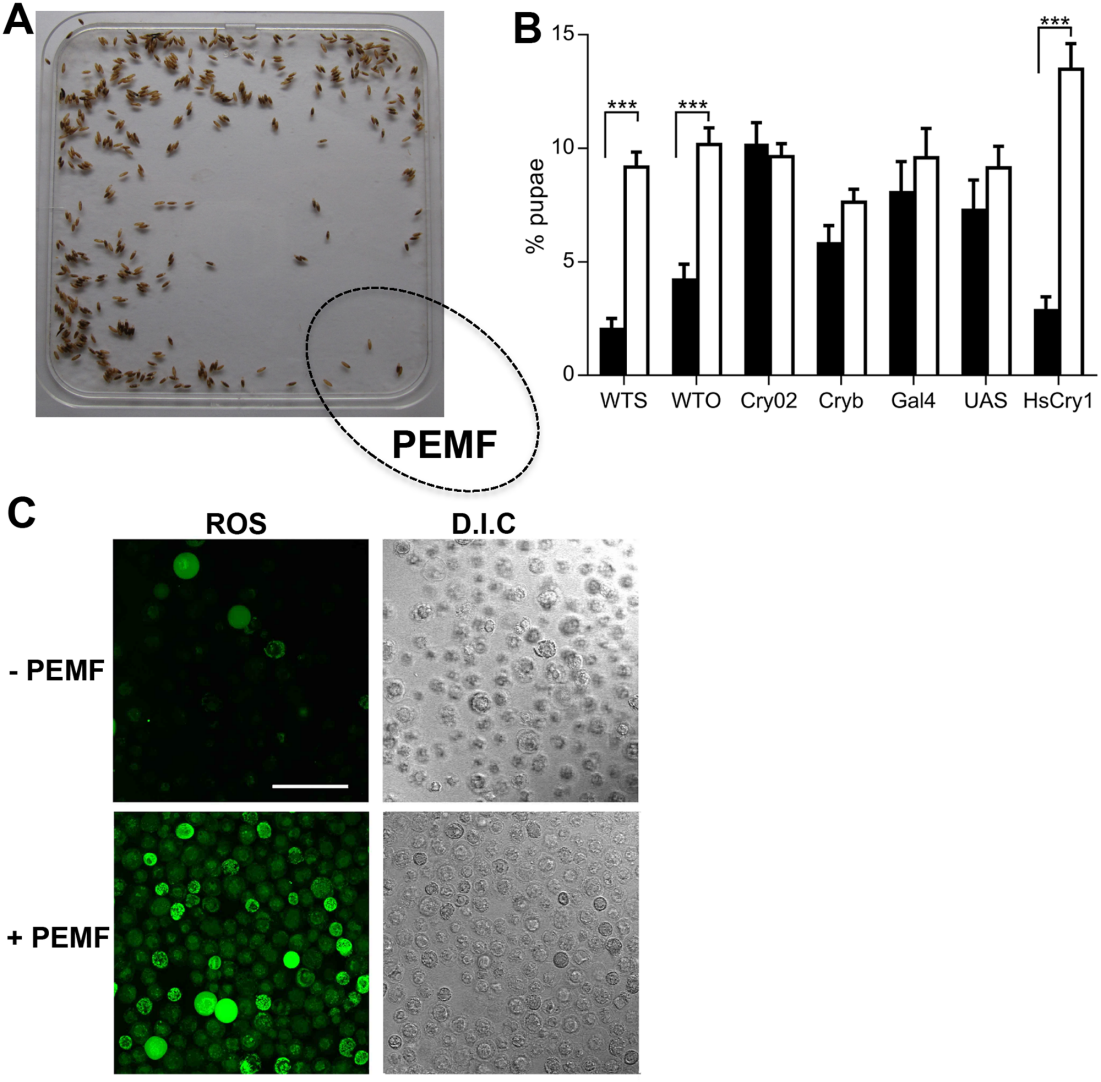
Insect behavioral and cellular response to PEMF. (A). Distribution of wild-type Canton S larvae following 96-hour exposure in blue light (60 μmolm^−2^s^−1^) to PEMF (10 Hz) to the indicated corner. (B) Response to PEMF expressed as percentage of larvae in the petri plate corners: PEMF exposed corner (black bars), mean of 3 nonexposed corners (white bars). Data represent averages from 8 to 10 independent biological experiments (*n* = 8–10) (see S1 Data). Strains used are Canton S (WTS), Oregon (WTO), cry-deficient mutants (*cry*^02^ and *cry*^b^). Gal4 and UAS are nonexpressing parental strains for the cross (*tim-gal4*;*cry*^02^ × *UAS-Hscry1*;*cry*^02^) (HsCry1) that expresses the HsCry1 protein as described in ref. [27]. ***p < 0.001 (see Materials and methods for details of statistical treatment). Error bars are SEM. (C) SF21 insect cells overexpressing DmCry exposed to PEMF. Dark grown Sf21 insect cell cultures expressing high concentrations of DmCry as described [28] were illuminated for 15 minutes at 80 μmolm^−2^s^−1^ blue light in the presence (+) or absence (−) of PEMF and were viewed by confocal microscopy as described in Materials and methods. *n* = 5 biological repeats. Scale bar 100 μm. Data for Fig 1B is in S1 Data. DmCry, *Drosophila* cryptochrome; Gal4, *tim-gal4*;*cry*^02^; HsCry1, human cryptochrome-1; PEMF, pulsed electromagnetic field; Sf21,; UAS, *UAS-Hscry1*;*cry*^02^.

To explore this question, we chose to use PEMF exposure as a source of magnetic stimulation because it has demonstrated therapeutic effects on a wide variety of mammalian cell types [5–10]. To determine whether cryptochromes are implicated in PEMF effects, we first established whether a known magnetosensitive cryptochrome can mediate a response to a PEMF signal in a well-established magnetically sensitive model system. We used the fruitfly *Drosophila melanogaster*, which display a natural behavioral avoidance response to static magnetic fields [16]. Adult flies were placed on square petri plates to lay eggs for 24 hours and were subsequently removed. The ensuing hatched larva migrated freely over the plate for several days before choosing a location to attach to and form sessile pupa for metamorphosis. These pupae were located randomly around the perimeter of the plate, with preference for the corners (Fig 1). We tested magnetic sensitivity, with a coil generating continuous PEMF at 10 Hz, with peak amplitude of 1.8 mT at the level of the larvae (S1 and S2 Figs), placed underneath one of the 4 corners of the petri plate (see Materials and methods). Fly larvae grown under these conditions avoided the corner of the petri plate above the PEMF device (Fig 1A) compared to the other corners. Both Canton S (WTS) and Oregon (WTO) wild-type fly strains showed this avoidance response (Fig 1A and 1B) in blue light (which activates *Drosophila* cryptochrome; Fig 1B) but not in red light (which does not activate *Drosophila* cryptochrome; S3 Fig). As a control, a 1.0 mm mu-metal plate, which blocks static or low-frequency magnetic fields, was inserted between the magnetic coil and the petri plate containing the fly larvae. In these conditions, larvae did not show the avoidance response (S3 Fig). As a further control, we tested a coil in which the wire had been wound in an antiparallel fashion in order to cancel the magnetic field without altering the current in any way (see Materials and methods); this was also ineffective in causing an avoidance response. We next observed that fly mutants deficient in cryptochrome (*cry*^b^ and *cry*^02^; [27]) did not avoid the PEMF, confirming a role for cryptochrome in this response. Finally, we tested transgenic fruitflies expressing the human cryptochrome-1 (HsCry1) protein in *Drosophila* cryptochrome-deficient strains as described previously [16, 27]). HsCry1 expression indeed restored the behavioral avoidance response to PEMF in flies lacking their endogenous cryptochrome (Fig 1B). These results indicate that PEMF can be detected by insects through the action of either *Drosophila* (DmCry) or human (HsCry1) cryptochrome, consistent with the response to static magnetic fields in this organism [16].

A possible mechanistic basis for this fly avoidance response was suggested by recent observations that ROS are byproducts of cryptochrome activation [17, 28] linked to signaling [29, 30]. Furthermore, at high concentrations, ROS are toxic metabolites implicated in oxidative stress and ageing, which damage cell membranes, nucleic acids, and proteins [20], consistent with the behavioral avoidance response. In contrast, at physiological concentrations, ROS are reported to have beneficial effects [20, 23], consistent with the observed therapeutic effects of PEMF [5–10]. To determine whether the PEMF signal stimulates formation of ROS, *Spodoptera frugiperda* (Sf21) insect cell cultures overexpressing DmCry [28] were stimulated by PEMF in blue light for 15 minutes in the presence of the ROS label, {5-(and-6)-chloromethyl-2’,7’-dichlorofluorecein diacetate} (DCFH-DA) [17, 28]. Confocal image analysis revealed a marked increase in fluorescent signal in PEMF-treated cells compared to unstimulated cultures (Fig 1C). In contrast, no visible effect of PEMF stimulation was observed in Sf21 cells lacking DmCry (S4 Fig). These data indicate that PEMF stimulation leads to intracellular accumulation of ROS and that this effect requires *Drosophila* cryptochrome.

**Fig 2:**
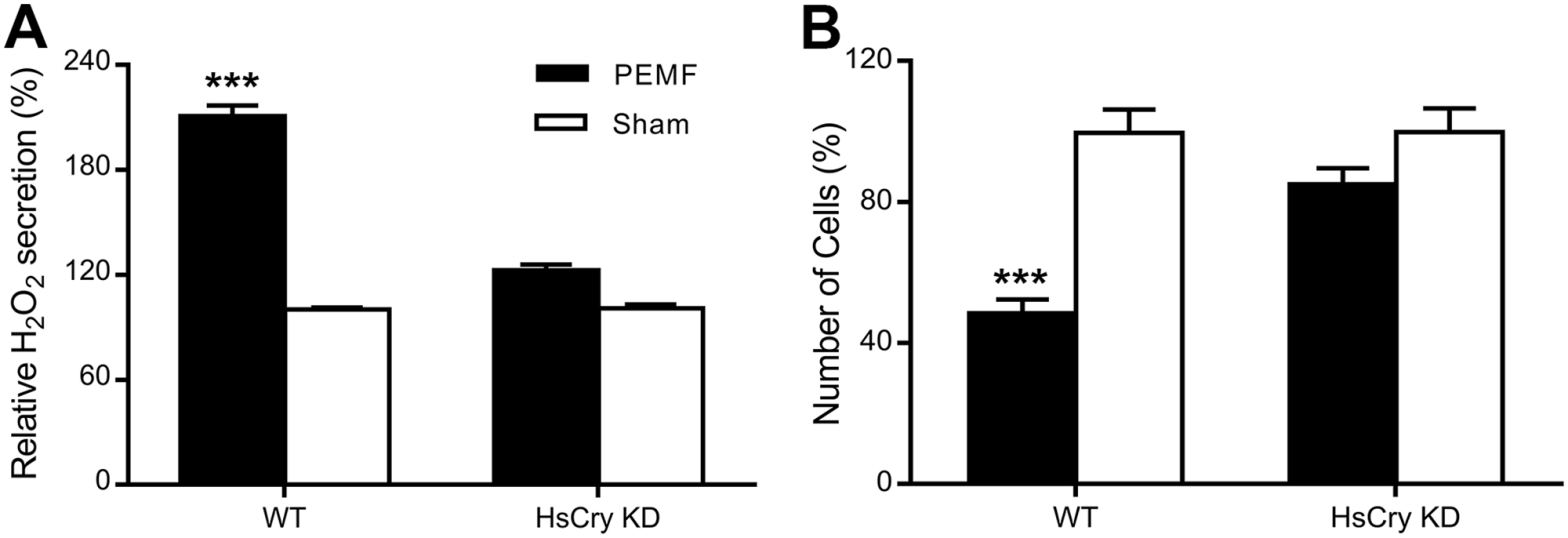
PEMF response of HEK293 cell cultures. (A) Cells of WT or *HSCRY1* and *HSCRY2* double KD (HsCry) were seeded in 24-well cell culture dishes and grown for 48 hours in the presence (PEMF, black bars) or absence (Sham, white bars) of applied PEMF as described (Materials and methods). The concentration of H_2_0_2_ in the culture media was determined using the Amplex Red fluorescent detection system (Materials and methods) and normalized to the number of cells. The graph presents the relative concentration of H_2_0_2_ from PEMF-treated cells (PEMF, black bars) compared to the control (Sham, white bars) untreated sample. Error bar represents SD of 12 independent measurements. (B) Relative number of cells per well in PEMF-treated cells (PEMF, black bars) compared to the control (Sham, white bars) untreated sample. *n* = 12 biological repeats. Error bar represents SD. ***p < 0.001 (see Materials and methods). Underlying data is included in S1 Data. HEK293, human embryonic kidney cells; HsCry, human cryptochrome; KD, knockdown; PEMF, pulsed electromagnetic field; WT, wild-type.

Although flavin binding affinity is reportedly poor for vertebrate cryptochromes in vitro [31], they nevertheless confer light-sensitive phenotypes in expressing transgenic flies [16, 27] and undergo light-sensitive conformational change in the avian retina [15, 32], indicating that flavin is bound in vivo. Moreover, vertebrate-type cryptochromes are shown to undergo photoreduction and flavin radical formation in whole cell cultures, using an EPR spectroscopic approach [33]. These properties are consistent with the capacity to undergo flavin redox state interconversion and to form ROS, as do other cryptochromes [17, 28, 34]. We therefore tested for ROS induction following PEMF stimulation of human embryonic kidney 293 (HEK293) cells, grown in darkness for 48 hours in the presence or absence of PEMF (Fig 2). After incubation, the extracellular media were scored for secreted hydrogen peroxide (H_2_O_2_), a byproduct of ROS formation, using the Amplex Ultra Red fluorescence detection substrate as described [35]. The concentration of ROS was significantly elevated in media from PEMF-treated cell cultures compared to controls (Fig 2). To evaluate toxicity of prolonged exposure to PEMF, we counted cells at the end of the exposure period (see Materials and methods). A marked decrease in cellular growth was observed in PEMF-exposed HEK293 cultures compared to untreated controls, consistent with the toxicity of accumulated ROS (Fig 2). To assess a possible effect of cryptochrome on this response, short hairpin RNA (shRNA) lines with double HsCry1 and HsCry2 mRNA knockdown were constructed (see Materials and methods, S5 Fig) and similarly analyzed. These shRNA lines deficient in both HsCry1 and HsCry2 showed no significant effect of PEMF either on cell growth or on ROS secretion (Fig 2A and 2B), in marked contrast to wild type. Therefore, these magnetic field effects appear to involve cryptochrome function and formation of ROS in human cells.

**Fig 3:**
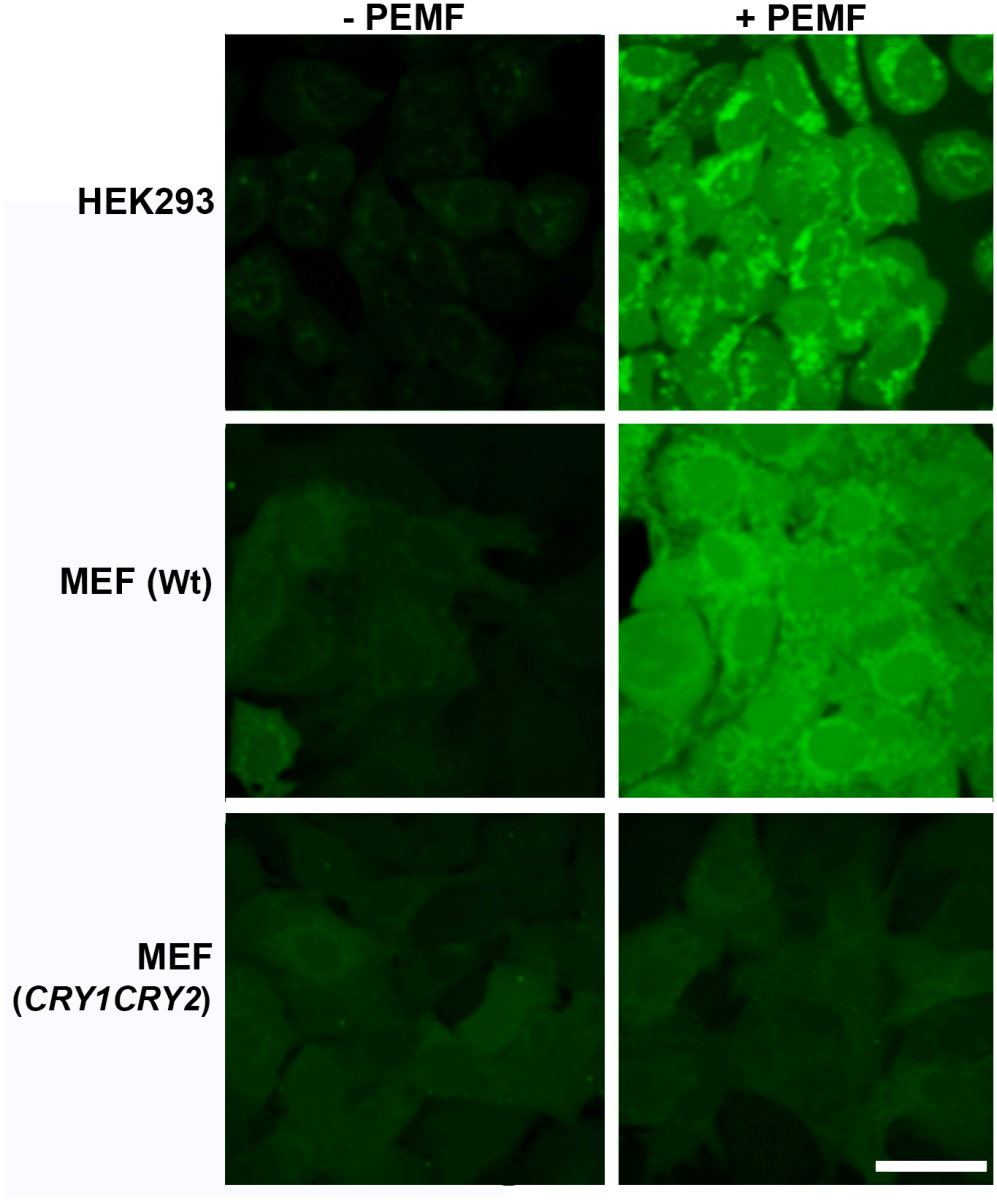
Production and subcellular localization of ROS by mammalian cells exposed to PEMF. Living HEK293, MEF, or MEF *CRY1CRY2* cryptochrome-deficient double mutant knockout cell lines were exposed to PEMF (+PEMF) for 15 minutes in darkness while simultaneously treated with DCFH-DA, then viewed by an inverted Leica TCS SP5 microscope. Control cell cultures not exposed to the magnetic field (−PEMF) were treated in an identical manner. Images show a single confocal z section that crosses the nucleus. Diffuse fluorescent ROS staining can be seen in the nucleus and cytoplasm. Punctate and intense fluorescent ROS staining colocalizes with ER and nucleoli, as observed around and inside the nucleus, respectively. Qualitatively similar results were obtained for 5 independent experiments (*n* = 5); quantitation of representative images is presented in S9 Fig. Scale bar is 40 μm. DCFH-DA, {5-(and-6)-chloromethyl-2’,7’-dichlorofluorecein diacetate}; ER, endoplasmic reticulum; HEK293, human embryonic kidney 293; MEF, mouse embryonic fibroblast; PEMF, pulsed electromagnetic field; ROS, reactive oxygen species.

We further analyzed PEMF effects on mammalian cells using fluorescence imaging to detect multiple ROS forms. As observed for the Sf21 insect cell experiments above (Fig 1C), HEK293 cells were incubated in the presence of DCFH-DA at 37 °C for 15 minutes in the presence or absence of PEMF (Fig 3). Fluorescent ROS labeling increased significantly in PEMF-stimulated cells compared to unstimulated control cell cultures. ROS staining can be seen in both nuclear and cytosolic compartments, with areas of concentration in nuclear speckles (nucleoli) and vesicular structures (E.R and Golgi), consistent with subcellular localization of mammalian cryptochromes [36].

To further confirm the involvement of cryptochrome in this response, we examined cells from murine cryptochrome mCry1/mCry2 double knockout mice [37]. Specifically, we analyzed immortalized mouse embryonic fibroblast (MEF) cell cultures from wild-type and mCry1/mCry2 double knockout lines, using the ROS fluorescence imaging techniques used for the HEK cell cultures. A marked induction of intracellular ROS after 15 minutes of PEMF stimulation was observed in wild-type MEF cells (Fig 3, middle panels), equivalent to those observed for the HEK293 human cell cultures (Fig 3, upper panels). However, mCry1/mCry2 null mutant cell cultures treated in an identical manner (Fig 3, lower panels) showed no visible increase in ROS labelling. Taken together, these data show that cryptochrome is necessary for PEMF-induced ROS formation in mammalian cells.

To further define the effects of PEMFs and relate them to therapeutic consequences observed in humans [5–10], we performed microarray analysis of gene expression in HEK293 cells cultured with or without 3 hours of PEMF stimulation (S1 and S2 Tables). Several hundred genes were up-regulated or down-regulated by PEMF stimulation. Of these transcripts, a significant proportion encoded proteins localized to nuclear, Golgi, and endoplasmic reticulum (ER) compartments (S5 Table). Significantly, bioinformatic gene ontology (GO) analysis of biochemical function showed enrichment in oxidoreductase function consistent with increased production of ROS (see Materials and methods, S6 Table). Furthermore, promoter analysis of PEMF-induced genes indicated that a majority (75%) contained promoter elements known to interact with ROS-responsive transcription factors. These data are consistent with stimulation of ROS-responsive genes following PEMF exposure (S7 Table). Furthermore, they parallel the imaging data of these HEK293 cells, which showed enhanced localization of ROS to the nuclear, Golgi, and ER compartments, whereas transcription of proteins localized to these compartments are particularly enriched among PEMF-regulated genes (S5 Table). Thus, the induction of ROS by PEMF is indicated by two entirely independent and complementary approaches: imaging and transcriptome analysis.

## Discussion

A widely held paradigm for cryptochrome magnetosensing involves a radical pair-based mechanism, whereby the singlet/triple interconversion rates of unpaired radicals formed in the course of cryptochrome redox chemistry can be altered by static magnetic fields [13]. This provides a mechanism whereby cryptochrome reaction rates and product yields, including of H_2_0_2_ and other ROS formed during the cryptochrome redox cycle [17,28], can be altered by magnetic fields. Recent experiments probing the light dependence of magnetic orientation in birds have pinpointed cryptochrome flavin reoxidation as the likely step for radical pair formation leading to magnetic sensitivity [32,38]. Such flavin reoxidation, which occurs independently of light, involves reaction of cryptochrome-bound reduced flavin with molecular oxygen and fulfills the criteria of radical pair formation during magnetoreception [39]. Nonetheless, in the case of both avian and drosophila cryptochromes, the initial formation of reduced flavin requires light (by the process of flavin photoreduction) [34,38]. This explains the requirement for light in establishing magnetic sensitivity in flies and birds because reduced flavin is required for the magnetically sensitive redox reaction (reoxidation) to ensue [38].

By contrast, mammalian-type cryptochromes appear to function independently of light in their role in the circadian clock and as negative regulators of transcription [14,24,25]. However, mammalian-type cryptochromes reportedly occur in a partially reduced redox state in vivo even in dark-adapted cell cultures [40]. As a consequence, they would retain the characteristics to respond to magnetic fields by a mechanism whereby flavin reoxidation is stimulated, with an ensuing burst of ROS synthesis consistent with our observations. We also note that, although there has been overwhelming evidence for a radical pair-based magnetic sensing mechanism involving vertebrate cryptochromes [13], the possibility of unrelated cry-dependent magnetosensing mechanisms cannot be excluded. For example, a recently suggested interaction of cryptochrome with the putatively magnetosensitive MagR protein could also be consistent with our data [41], whereas reported magnetic sensitivity mediated through a C-terminal overexpression construct of *Drosophila* cryptochrome [42] also suggests alternative magnetosensors impacting on a cry-based magnetosensing mechanism.

A mechanism based on regulation of ROS can explain both the beneficial and deleterious effects of magnetic stimulation that have so long puzzled the field. For example, proposed deleterious effects [1–4,26] of low-frequency EMFs could result from elevated ROS, which inform about exposure to magnetic fields either in human treatment or in public health. This result is furthermore consistent with past suggestions that the lifetimes and reactivity of 0_2_ and ROS (both paramagnetic species) may be affected by magnetic fields in living systems [5]. However, prior speculation has focused exclusively on ROS generated via metabolic pathways of the mitochondrial electron transfer chain or via cell membrane–associated NADPH oxidases. Here, we implicate a flavoprotein receptor and signaling molecule, which is suitably positioned within the nucleus [36], to induce localized changes in ROS concentration and/or reactivity in close proximity to redox-sensitive and/or ROS-regulated nuclear signaling molecules. We note that the prolonged PEMF signal (S1 and S2 Figs) used in the current study has no therapeutic application and is apparently harmful to cell cultures over long periods. However, a range of alternate frequencies and amplitudes of PEMF signal have been empirically derived that provide proven physiological benefits involving cellular repair and healing [5–12]. These beneficial PEMF effects are compatible with modulation of intracellular ROS within a therapeutic range resulting in stimulation of ROS responsive cellular defense and repair mechanisms [20,23].

In conclusion, from a public health perspective, our work shows that exposure to even such low levels of magnetic fields as those generated by PEMF devices have definite physiological consequences. It should be noted that peak output at less than 1.8 mT is within an order of magnitude of emissions by household electronic devices and of current safety guidelines for exposure to EMF in humans [1–4]. In keeping with our results, it has also been shown that the low-level man-made EMFs emitted from electrical equipment in public buildings can disrupt orientation in birds, a process that has also been linked to both cryptochromes and magnetoreception [43]. Although current epidemiological studies have not provided conclusive evidence of EMF-induced pathology in humans [1–4], our results raise the possibility of synergistic harmful effects with other environmental or cellular factors that stimulate intracellular ROS [5,20]. More refined epidemiological studies taking these factors into consideration are therefore essential for a true assessment of long-term impact of EMFs on public health.

## Materials and methods

### 1. Pulsed EMF signal

The pulsed magnetic field was generated by a commercially available device (EC10701; GEM Pty Ltd., Perth, Western Australia) used for the treatment of musculoskeletal disorders. During *Drosophila* behavioral tests, PEMF was applied continuously at a frequency of 10 Hz, with the coil 1 cm below the petri plate. Peak magnetic intensity at the experimental distance was 2 mT.

The parameters of the PEMF signal were verified by measurement of the current as presented in S1 and S2 Figs. The coil was 9 × 5.5 cm and 200 turns and produced a maximum magnetic field intensity 1 cm above the coil of 1.8 mT.

### 2. Drosophila behavioral experiments

Fly strains used were as follows: wild-type Canton S, wild-type Oregon-R, *cry*^02^ and *cry*^b^ (described in [27]). Transgenic strains *tim-gal4*;*cry*^02^ and *UAS-Hscry1*;*cry*^02^ were crossed to generate the heterozygote HsCry1-expressing strain as described in Vieira and colleagues [27]. Light sources and growth conditions on complete media were as previously described [27]. For the larvae migration studies (pupal distribution), adult drosophila were transferred to a square plate (12.5 cm × 12.5 cm) containing complete rich medium and were allowed to lay eggs. After a period of 24 hours, the adults were discarded and the plates placed under the indicated light conditions (blue or red light) for 3 days at 23 °C. Subsequently, a single corner of each plate was exposed to PEMF from underneath for an additional 5 days. Temperature differential between corners was less than 0.5 °C. The control condition was established by shielding the plate from the PEMF device with 1.0 mm mu-metal sheeting, which was measured to reduce magnetic field signal by 85%. Once pupal development was complete, the distribution of the now nonmotile pupae could be readily scored by counting the number of pupae in a defined area of the plate. As the pupae showed a preference for the corners of the plates, we evaluated 3.12 × 3.12 cm^2^ areas over each of the 4 (PEMF treated versus untreated) corners and compared the corners that had received no PEMF treatment with those exposed to PEMF. Statistical methods were as follows: for each experimental condition, a total of between 8 and 10 plates were analyzed (*n* = 8–10). The number of drosophila counted in each corner was expressed as the percentage of the total number of pupae ± SEM per plate. All statistical tests were carried out using SPSS (version 20, IBM Corporation, NY). Data were analyzed for normality (Shapiro-Wilk test, *p* < 0.05), so the differences between the PEMF and mean of the 3 non-PEMF corners per plate were compared using Kruskal-Wallis analysis of variance and Mann-Whitney-U post hoc where appropriate. The α value was set to *p* < 0.05.

Further details of the behavioral experimental setup are presented in S3 Fig as follows: the position of the plate containing drosophila growth media and growing larva under which the PEMF coil was placed (upper left) is designated as the position “1,” the “test” corner. The PEMF coil was at a distance of 1 cm from the bottom of the test plate containing the drosophila larvae. The temperature at all 4 corners was measured, and the PEMF device did not cause any change in temperature from the other corner positions of the plate. Equivalent-size squares at each of the other corners (designated positions 2, 3, and 4) serve as the internal “control” positions to the PEMF stimulated “test” position. The number of pupae that were deposited beneath the PEMF coil was compared to the number of pupae deposited within an equivalent volume at each of the other 3 corner positions.

Additional controls to the behavioral experiments are shown in S3B and S3C Fig. The PEMF device was shielded from the test plate using mu-Metal sheeting of 1.0 mm thickness. Under these conditions (S3B Fig), no significant avoidance of the PEMF corner position was detected. In addition, response to PEMF was scored in red light, which does not activate insect (*Drosophila*) cryptochrome (S3C Fig). In this case also, no avoidance of the PEMF was observed.

### 3. Intracellular localization of ROS in DmCry-expressing insect cell cultures

Preparation of DmCry-expressing and control (Spa1)-expressing insect cell cultures was performed as described in Arthaut and colleagues [28]. For imaging experiments, Sf21 cells were seeded at a density of 400,000 cells in a 3.5 cm^2^ observation chamber. After incubation at RT for 2 hours for cell attachment, Sf21 cells were incubated in 40 mM potassium phosphate buffer (pH 6.4) containing 12.5 μM DCFH-DA (Molecular Probes, Life Technologies, Grand Island, NY) for 15 minutes in the dark, rinsed 2 times in phosphate buffer, and were then exposed to blue light with or without PEMF for 15 minutes and observed with an inverted Leica TCS SP5 confocal microscope using a 40× objectif. Green fluorescence from DCFH-DA and differential interference contrast (DIC) were excited at 488 and 561 nm wavelengths, respectively. Emission fluorescence intensities and DIC were detected using a photomultiplier between 498 and 561 nm, and a transmission photomultiplier, respectively. Two channels were recorded sequentially. Z series projections were taken using ImageJ software (W. S. Rasband, ImageJ). As a control for these experiments, control cell cultures that did not express DmCry were used (S4 Fig). These cells did not show induction of ROS in response to PEMF.

### 4. Construction of human *CRY1-* and *CRY2*-targeting shRNA plasmids and Hscry1/Hscry2 knockdown HEK cells

By using the InvivoGen siRNA Wizard tool, shRNA sequences targeting human CRY1 (NM_004075.4) and CRY2 (NM_021117.3) were selected, and a pair of complementary (sense and antisense) oligonucleotides were designed for each sequence as follows:

shCRY1 sense (5’GTACCTCGGAACGAGACGCAGCTATTAATCAAGAGTTAATAGCTGCGTCTCGTTCCTTTTTGGAAA 3’); shCRY1 antisense (5’AGCTTTTCCAAAAAGGAACGAGACGCAGCTATTAACTCTTGATTAATAGCTGCGTCTCGTTCCGAG3’); shCRY2 sense (5’ACCTCGTACGTATGTCACCTTCACTATCAAGAGTAGTGAAGGTGACATACGTACTT3’); shCRY2 antisense (5’CAAAAAGTACGTATGTCACCTTCACTACTCTTGATAGTGAAGGTGACATACGTACG3’) (complementary sequences of the hairpin are underlined). Complementary oligonucleotide pairs were PAGE-purified, and 25 μM of each were annealed by incubation in 0.1 M NaCl at 80 °C (2 minutes) followed by slow (1 °C per minute) cooling to 35 °C. The resulting double-stranded DNA fragments were cloned into the same psiRNA-DUO-GFPzeo plasmid according to the manufacturer’s instructions using a two-step procedure (InvivoGen). Briefly, the psiRNA-DUO plasmid was digested with Acc65I and HindIII restriction enzymes and ligated with the first insert (shCRY1 annealed oligonucleotide pairs). The resulting construct was transformed into *Escherichia coli* GT115 cells (InvivoGen), and positive colonies were selected using Fast-Media Zeo X-gal (5-bromo-4-chloro-3-indolyl-β-d-galactopyranoside) (InvivoGen). The plasmid containing shCRY1 was subsequently digested with BbsI restriction enzyme and ligated with the second insert (shCRY2 annealed oligonucleotide pairs). The resulting construct was transformed into *E*. *coli* GT115 cells (InvivoGen), and positive colonies were selected using Fast-Media Zeo 5-bromo-4-chloro-3-indolyl-β-D-glucuronic acid, cyclohexylammonium salt (X-gluc) (InvivoGen). The obtained psiRNA-Cry1Cry2-GFPzeo expression plasmid was used for transfection of HEK cells, and psiRNA-LucLac-GFPzeo encoding shRNA for the silencing of a prokaryote gene (InvivoGen) was used to transfect HEK cells as nonsilencing control. Stable transfectants were selected in complete medium containing 300 μg/ml Zeocin (InvivoGen). Expression of HsCry1 and HsCry2 was verified by qPCR (S5 Fig).

### 5. Fluorimetric detection of H_2_0_2_ in HEK cell culture medium

HEK293 cells were grown and maintained in Eagle’s Minimum Essential Medium (EMEM), supplemented by 10% fetal bovine serum. The cells were cultured in 75 cm^2^ flasks to expand cell number. After reaching confluence, the cells were seeded in 12-well plates. The volume of medium totaled 1 mL. Medium was then changed every 2 days. The cultures were incubated in a 5% CO_2_ atmosphere at 37 °C in the same incubator (Fisher Scientific; Model 5). The temperature and CO_2_ levels were monitored daily and were maintained at 37 °C and 5%, respectively. All experiments were conducted in the same incubator. To control for location in the incubator and any associated electromagnetic noise or other spatial variation, the orientation of experimental and control cultures were periodically reversed, and 0.3 mm mu-Metal shielding was applied between PEMF-treated and control cell culture dishes within the incubator. Cells were seeded and allowed to rest for 4 hours under the same background conditions, at which time the magnetic exposures began. This time is denoted as t_0_. Fluorometric detection of H_2_O_2_ production was performed using the horseradish peroxidase-linked Amplex Ultra Red (Invitrogen) fluorometric assay. HEK cells were seeded at a concentration of 25.0 × 10^4^ cells per well in a 12-well plate and were exposed to PEMFs for the duration of the experiment. Medium was aspirated off, and cells were then washed with PBS and incubated for 2 hours with DMEM containing 2% FBS, 0.2 units/ml horseradish peroxidase, and 10 μM Amplex UltraRed (AUR). Resorufin fluorescence was measured by a Varian Cary Eclipse spectrofluorimeter. Cellular number and resorufin fluorescence were measured at the same termination points. H_2_O_2_ production was normalized to cell count. H_2_O_2_ calibration curves with HRP-AUR in PEMFs did not show any difference compared to control, thus demonstrating that PEMFs do not interact with the detection system.

### 6. Intracellular localization of ROS in human HEK293 or murine MEF cell cultures using confocal imaging techniques

Human HEK and MEF cells were grown in Dulbecco’s Modified Eagle Medium (DMEM) supplemented with 10% fetal calf serum (FCS) and 2 mM l-glutamine in a 95% air–5% CO_2_ incubator at 37 °C. For intracellular localization of ROS, living HEK or MEF cells were seeded on cell observation chambers and incubated in 40 mM potassium phosphate buffer (pH 7) containing 12.5 μM DCFH-DA (Molecular Probes) for 15 minutes in the incubator at 37 °C, during which they were either exposed or not to PEMF. Cells were rinsed for 15 minutes in the potassium phosphate buffer solution and were observed with an inverted Leica TCS SP5 confocal microscope equipped with a 95% air–5% CO_2_−37 °C thermostatic observation chamber and using a 63× objective. Green fluorescence from DCFH-DA and DIC were detected as previously described (section 5). For quantitation of intensity, using LEICA TCS software, the region of interest (ROI) corresponding to cells were dawn and mean fluorescent intensity (MFI) measured in each ROI.

### 7. Microarray analysis of HEK293 cell cultures

Human HEK cells were grown in DMEM supplemented with 10% FCS and 2 mM l-glutamine in a 95% air–5% CO_2_ incubator at 37 °C. Cells were seeded into multiple 3.5 cm^2^ round cell culture dishes and were grown under identical conditions for 48 hours. Prior to confluence, cell culture dishes were treated with 3 hours of continuous PEMF in the absence of light (test condition). Control cell cultures were harvested prior to application of PEMF. Triplicate PEMF treated and control cell cultures were then harvested into liquid nitrogen, and total RNA was extracted by RNEasy RNA extraction kit (Promega, Inc.) and related protocols. Microarray gene expression and analysis using Agilent affymatrix technology was performed by IMGM Laboratories GmbH, Martinsried, Germany.

#### 7.1 Transcriptional analysis of altered gene expression in response to PEMF exposure of HEK293 cell cultures

Genes induced and repressed after exposure of HEK293 cells to 3 hours of PEMF identified 488 up-regulated (S1 Table) and 80 down-regulated (S2 Table) transcripts, respectively. Microarray expression data for representative up- and down-regulated genes were verified by qPCR analysis as presented in S6 Fig, using primers as presented in S3 Table.

For biological interpretation of the transcriptome dataset, the significantly overrepresented GO terms, biological pathways, and transcription factor binding sites (TFBSs) were explored with the R (3.3.0) and the Bioconductor suite (3.3). GO, pathways, and TFBS were considered significantly overrepresented with an FDR < 0.05.

GO analysis of biological function (S4 Table) shows enrichment of enzymes involved in cyclic nucleotide metabolism, corticosteroid receptor pathway, and wound healing; GO analysis of subcellular localization shows enrichment in Golgi, vesicles, ER, and nucleolar compartments (S5 Table); and GO analysis of biochemical function shows enrichment in oxidoreductase function and cyclic nucleotide metabolism (S6 Table). These functions show consistency with ROS induction and localization data presented in Figs 2 and 3 in the main text.

#### 7.2 Enrichment of ROS-responsive elements in promoters of PEMF-regulated genes

Because PEMF is shown to induce accumulation of ROS, we additionally tested whether PEMF-induced genes could be linked to ROS signaling pathways. We analyzed 248 PEMF–up-regulated genes with known functions (from S1 Table) for the presence of ROS response elements in their promoter regions using bioinformatics methodology as described in Beel and colleagues [44]. The number and type of ROS-responsive elements were identified in 2 kb of promoter regions upstream of the transcriptional start sites of PEMF-regulated genes (S7 Table). We observed that the majority (over 75%) of analyzed PEMF-regulated genes showed one or more ROS-responsive upstream promoter element (S7 Table). The transcriptional analysis data therefore support a role for PEMF in inducing ROS biosynthesis and signaling pathways.

### 8. Cancelled magnetic field coil control experiments

As a control to eliminate the possibility of artifact due to temperature and/or vibrational factors generated by the pulsed field device, we designed and built a modified PEMF coil in which the wire was folded in half before precision winding to achieve an antiparallel current travelling in opposite directions within the same coil during activation. This antiparallel coil had the same wire length and dimensions as the test coil used in our experiments, and it was driven by the same pulsed field generator device and with the identical current—which, because of the antiparallel winding of the coil, ran simultaneously in opposing directions within the coil. The signal measured in S7 Fig shows that, whereas there are residual spikes in the antiparallel field coil (panel B) that could not be cancelled, these spikes are less than 0.01 seconds in duration in comparison to the magnetic signal in the original PEMF coil, which lasts for 0.5 seconds (panel A). The residual spikes are not visible on panel D because they are too short lived for the detection limit for the instrument at this time scale. We conclude that these residual spikes are of negligible duration compared to the signal given out by the intact coil and are demonstrably too brief to trigger a biological response. As a result, we achieved a significant reduction (cancelling) of the pulsed magnetic field (S7 Fig) while keeping all other parameters (electric current driven by the pulsed field device) the same.

We tested the effect of the cancelled PEMF field on the behavioral avoidance response of wild type (Canton S) fly pupa according to the methods used for Fig 1. We measured the number of pupa in the corner of square petri plates exposed to the antiparallel (cancelled PEMF field) coil compared to the test coil (generating the PEMF signal) placed beneath the plate corner (S8 Fig). The flies did not show an avoidance response to the cancelled field (antiparallel coil), indicating that the magnetic field of the PEMF was indeed triggering the response.

We next evaluated the effect of the cancelled magnetic field coil on the stimulation of ROS in mammalian cell culture experiments. Using both HEK and MEF cell cultures, we observed that significant stimulation of ROS formation occurred only in response to PEMF but not to the antiparallel, PEMF-cancelled magnetic field (S9 Fig).

In conclusion, these data indicate that there was no discernable artifact introduced into our experiments through the operation of the pulsed field device and that positive results required the presence of the magnetic field.

## Supporting information

S1 Fig Output of PEMF device. Output was measured with a current probe directly connected to EC10701 stimulator. Current I as function of time.
(TIF)

S2 Fig Zoom of S1 Fig. Output of PEMF during a pulse. (TIF)

S3 Fig *Drosophila* pupal distribution in response to PEMF. Black bar: exposed corner; white bar: nonexposed corners. (a) Diagram of experimental setup showing the position of PEMF coil (upper left), as the “test corner.” The other corner positions of equivalent volume (designated positions 2, 3, and 4) serve as the internal “control” positions. (b) Distribution of pupae in response to PEMF in red light (60 μmolm^−2^sec^−1^). Strains used are wild-type strains Canton S (WTS) and Oregon (WTO), and cry-deficient mutants (*cry*^02^ and *cry*^b^). Black bar represents the exposed corner; white bar represents the nonexposed corner. (c) Drosophila strains exposed to PEMF under blue light (60 μmolm^−2^sec^−1^) with a 1.0 mm mu-metal sheet placed between the PEMF device and the bottom of the petri plate containing the flies (at position 1). Black bars represent the exposed corner; white bars represent the nonexposed corner. Strains used are Canton S (WTS), Oregon (WTO), cry-deficient mutants (*cry*^02^ and *cry*^b^). Gal4 and UAS are nonexpressing parental strains for the cross (*tim-gal4*;*cry*^02^ × *UAS-Hscry1*;*cry*^02^) (HsCry1) that expresses the HsCry1 protein as described in ref. [27]. Error bars are SEM. Underlying data for graphs b and c are in S2 Data. Gal4, *tim-gal4*;*cry*^02^; UAS, *UAS-Hscry1*;*cry*^02^.
(TIF)

S4 Fig Effect of PEMF on Sf21 ROS production in control cell cultures. SF21 insect cells expressing a nonphotoreceptor control protein SPA1 [28] in the absence of DmCry1 were illuminated for 15 minutes at 80 μmolm^−2^sec^−1^ blue light in the presence (+) or absence (−) of PEMF and viewed by confocal microscopy as described in [28]. No difference in ROS staining was observed. *n* = 5 independent biological replicates. Scale bar 100 μm.
(TIF)

S5 Fig qPCR analysis of *HSCRY1* and *HSCRY2* gene expression in antisense HEK293 cell lines. *HSCRY1* and *HSCRY2* gene expression is shown in control cells harboring the psiRNA-DUO-GFPzeo plasmid without shRNA insert (black bars) and compared to shRNA lines containing antisense constructs to *HSCRY1* and *HSCRY2* genes (HsCry KD: grey bars) constructed as described above. Primers used for qPCR analysis were as follows: HsCry1 Forward: 5’-GTGTTTCCCAGGCTTTTCAA-3’; HsCry1 Reverse: 5’-TGGTTCCATTTTGCTGATGA-3’; HsCry2F: 5-CTCGGAACAGTGCCTCAAATC-3; HsCry2 R: 5-GATAACGACCCTTCCACACAA-3. Data used to create graphs are in S2 Data.
(TIF)

S6 Fig qPCR analysis of gene expression in response to 3 hours of stimulation by PEMF in HEK293 cell cultures. PEMF-treated (black bars) are compared with sham-treated (white bars) cells. Primers used and designation of accession numbers are described in S1 Table. Underlying data are in S2 Data.
(TIF)

S7 Fig Comparison of output of PEMF and cancelled PEMF coil in mT as a function of time. Panels A and B: magnetic field output is on a millisecond (ms) time scale. Shape of signal in the original coil (panel A) is compared to that in the cancelled PEMF coil (panel B). Note the exceedingly short (less than 0.01 ms) duration of the spike in the cancelled field condition compared to signal of the original coil (0.5 msec duration). Panels C and D are on a slower (second) time scale. Panel C represents the zoom out of signal in panel A from the PEMF coil. Panel D represents the zoom out of the signal from the cancelled coil in panel B. The signal is too short to be detected in the cancelled coil at this time scale.
(TIF)

S8 Fig Response of wild-type (Canton S) fly larvae to PEMF delivered by normal (black bar) or antiparallel (grey bar) coils. The percentage of pupae shown is that in the exposed petri plate corners as a percentage of pupae in all corners (see Materials and methods for full description of experimental procedure and analysis). The flies showed avoidance of corners exposed to pulsed magnetic field signal (see S1 Fig) but did not show avoidance to a cancelled PEMF signal using an antiparallel coil with cancelled magnetic field (see S7 Fig). The horizontal dotted line is 25%, i.e., the percentage of pupae present by chance, and the percentage in the PEMF corner is significantly reduced (MWU, *p* = 0.028). *n* = 4 independent biological experiments; error bar is SEM. Underlying data are in S2 Data.
(TIF)

S9 Fig Production and subcellular localization of ROS by mammalian cells exposed to PEMF and control antiparallel coil. Living HEK293 or MEF were exposed either to PEMF (+PEMF) or cancelled PEMF (see S7 Fig for signal) for 15 minutes in darkness, simultaneously treated with DCFH-DA, then viewed by an inverted Leica TCS SP5 microscope. Control cell cultures (−PEMF) were treated in an identical manner but not exposed to either parallel or antiparallel PEMF coils (no exposure to any electrical or magnetic field). Images show a projection of all confocal z section. Scale bar 40 μm. Quantification of PMF effect is indicated by MFI ratio for each cell line (see Materials and methods). *n* = 5 independent biological repeats for all conditions. DCFH-DA, {5-(and-6)-chloromethyl-2’,7’-dichlorofluorecein diacetate}.
(TIF)

S1 Table Genes up-regulated by PEMF in HEK cell culture. (XLSB)

S2 Table Genes down-regulated by PEMF in HEK cell culture. (XLSB)

S3 Table Table showing the 9 genes selected for qPCR analysis (in S6 Fig) and their designed primers. (XLSX)

S4 Table GO biological function analysis of PEMF-regulated genes. (XLS)

S5 Table GO subcellular localization analysis of PEMF-regulated genes. (XLS)

S6 Table GO biochemical function analysis of PEMF-regulated genes. (XLS)

S7 Table Analysis of PEMF-regulated genes for ROS-responsive upstream promoter elements. (XLSX)

S1 Data Data files for Figs 1 and 2. (XLSX)

S2 Data Data files for Supporting S3b, S3c, S5, S6 and S8 Figs. (XLSX)

## Abbreviations

AUR: Amplex UltraRed
DCFH-DA: {5-(and-6)-chloromethyl-2’,7’-dichlorofluorescein diacetate}
DIC: differential interference contrast
DmCry: *Drosophila* cryptochrome
DMEM: Dulbecco’s Modified Eagle Medium
EMEM: Eagle’s Minimum Essential Medium
EMF: electromagnetic field
ER: endoplasmic reticulum
FCS: fetal calf serum
GO: gene ontology
HEK293: human embryonic kidney cells
HsCry1: human cryptochrome-1
MEF: mouse embryonic fibroblast
MFI: mean fluorescent intensity
PEMF: pulsed electromagnetic field
ROI: region of interest
ROS: reactive oxygen species
Sf21: Spodoptera frugiperda
shRNA: short hairpin RNA
TFBS: transcription factor binding site
