# Supplementary figures and images for: Low-intensity electromagnetic fields induce human cryptochrome to modulate intracellular reactive oxygen species
Source: PLoS Biol. 2018 Oct 2;16(10):e2006229. doi: 10.1371/journal.pbio.2006229 (PMC6168118; doi:10.1371/journal.pbio.2006229)

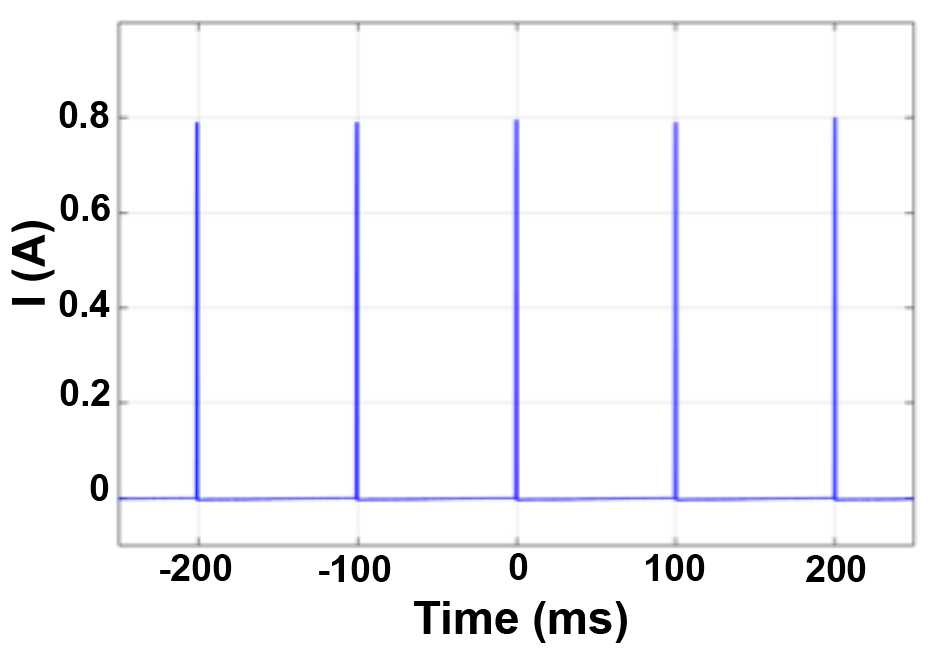

Supplement: S1 Fig — Output was measured with a current probe directly connected to EC10701 stimulator. Current I as function of time. (TIF) [file pbio.2006229.s001.tif]

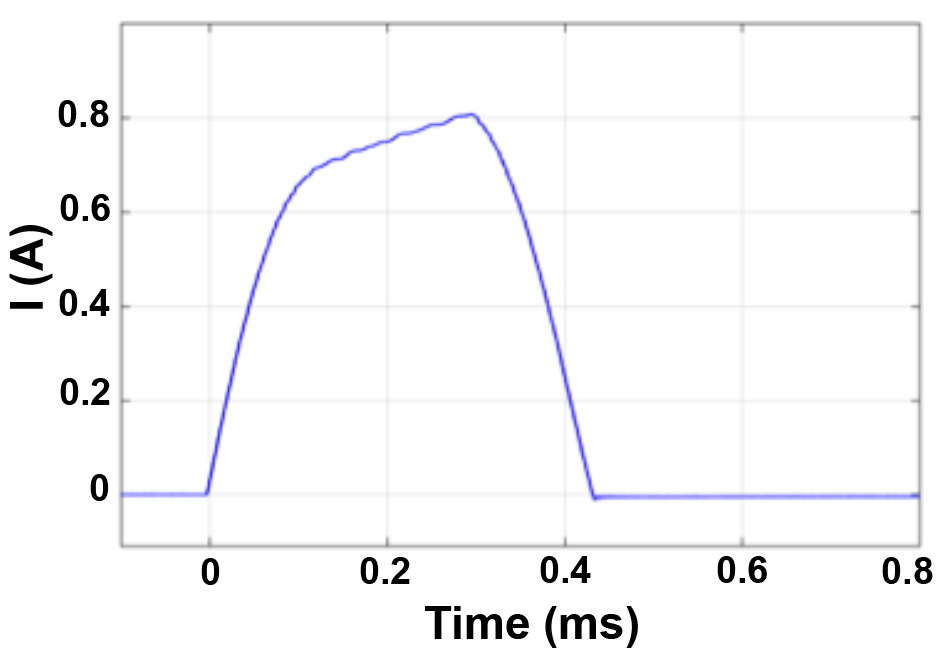

Supplement: S2 Fig — (TIF) [file pbio.2006229.s002.tif]

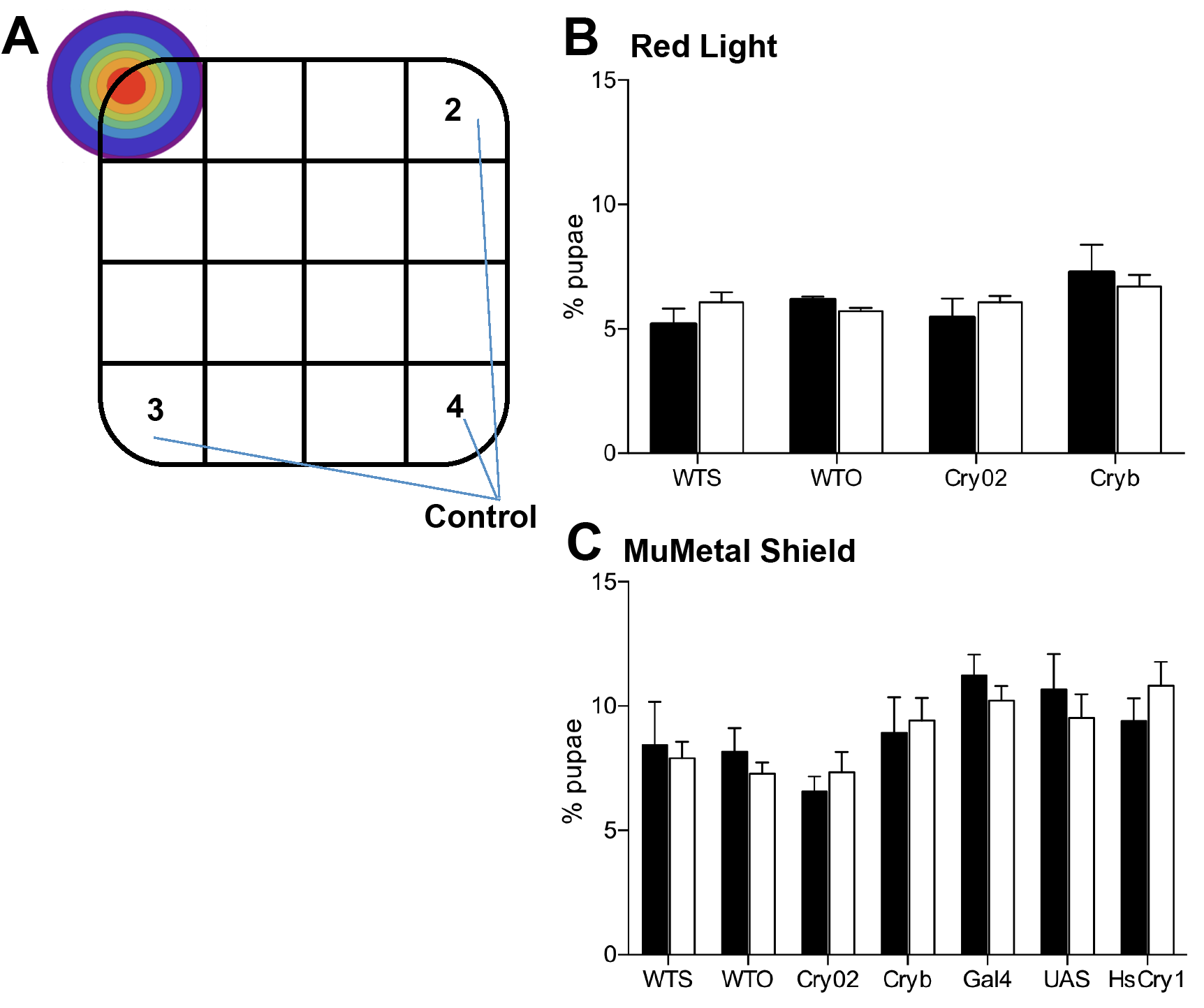

Supplement: S3 Fig — Black bar: exposed corner; white bar: nonexposed corners. (a) Diagram of experimental setup showing the position of PEMF coil (upper left), as the “test corner.” The other corner positions of equivalent volume (designated positions 2, 3, and 4) serve as the internal “control” positions. (b) Distribution of pupae in response to PEMF in red light (60 μmolm−2sec−1). Strains used are wild-type strains Canton S (WTS) and Oregon (WTO), and cry-deficient mutants (cry02 and cryb). Black bar represents the exposed corner; white bar represents the nonexposed corner. (c) Drosophila strains exposed to PEMF under blue light (60 μmolm−2sec−1) with a 1.0 mm mu-metal sheet placed between the PEMF device and the bottom of the petri plate containing the flies (at position 1). Black bars represent the exposed corner; white bars represent the nonexposed corner. Strains used are Canton S (WTS), Oregon (WTO), cry-deficient mutants (cry02 and cryb). Gal4 and UAS are nonexpressing parental strains for the cross (tim-gal4;cry02 × UAS-Hscry1;cry02) (HsCry1) that expresses the HsCry1 protein as described in ref. [27]. Error bars are SEM. Underlying data for graphs b and c are in S2 Data. Gal4, tim-gal4;cry02; UAS, UAS-Hscry1;cry02. (TIF) [file pbio.2006229.s003.tif]

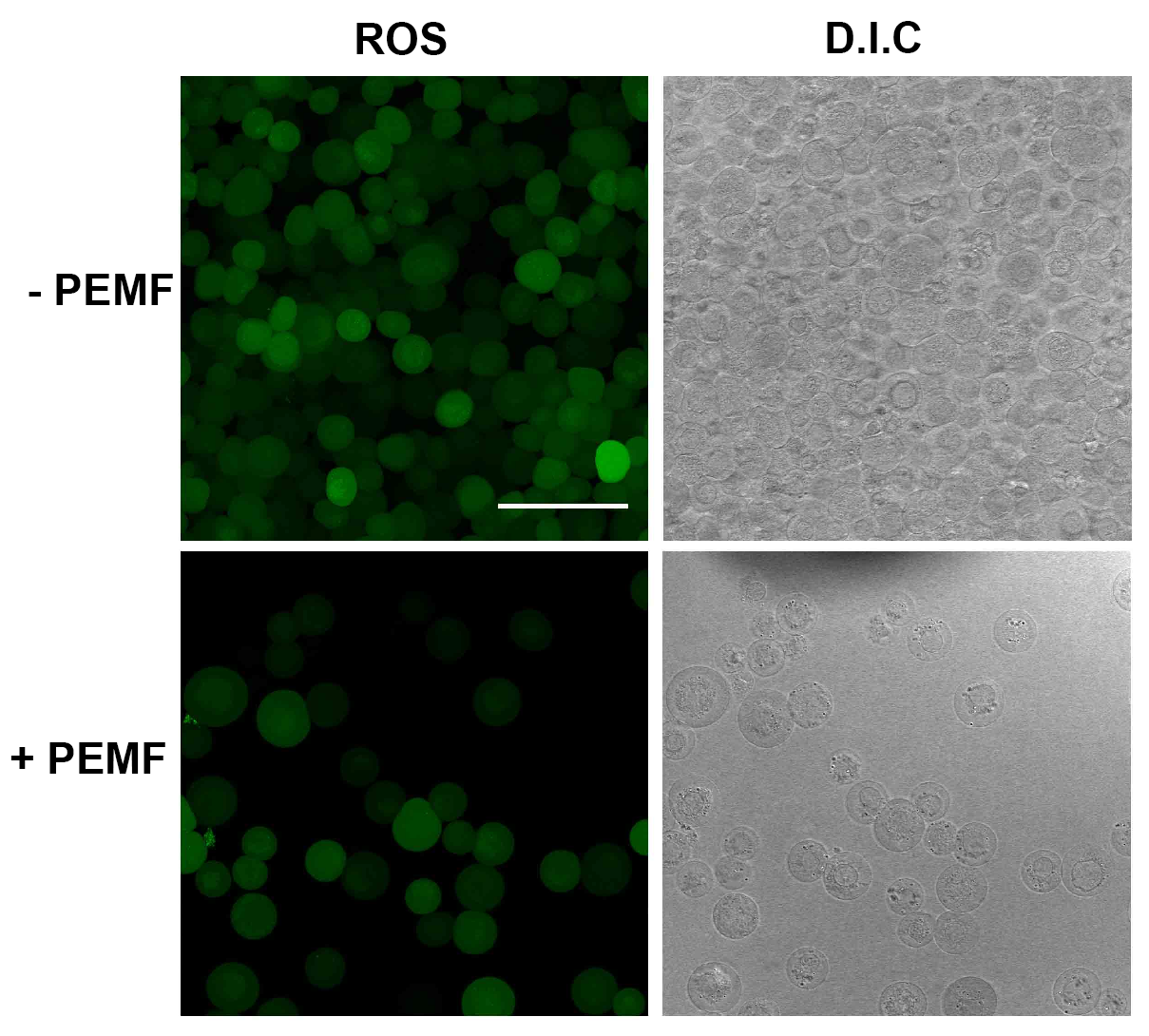

Supplement: S4 Fig — SF21 insect cells expressing a nonphotoreceptor control protein SPA1 [28] in the absence of DmCry1 were illuminated for 15 minutes at 80 μmolm−2sec−1 blue light in the presence (+) or absence (−) of PEMF and viewed by confocal microscopy as described in [28]. No difference in ROS staining was observed. n = 5 independent biological replicates. Scale bar 100 μm. (TIF) [file pbio.2006229.s004.tif]

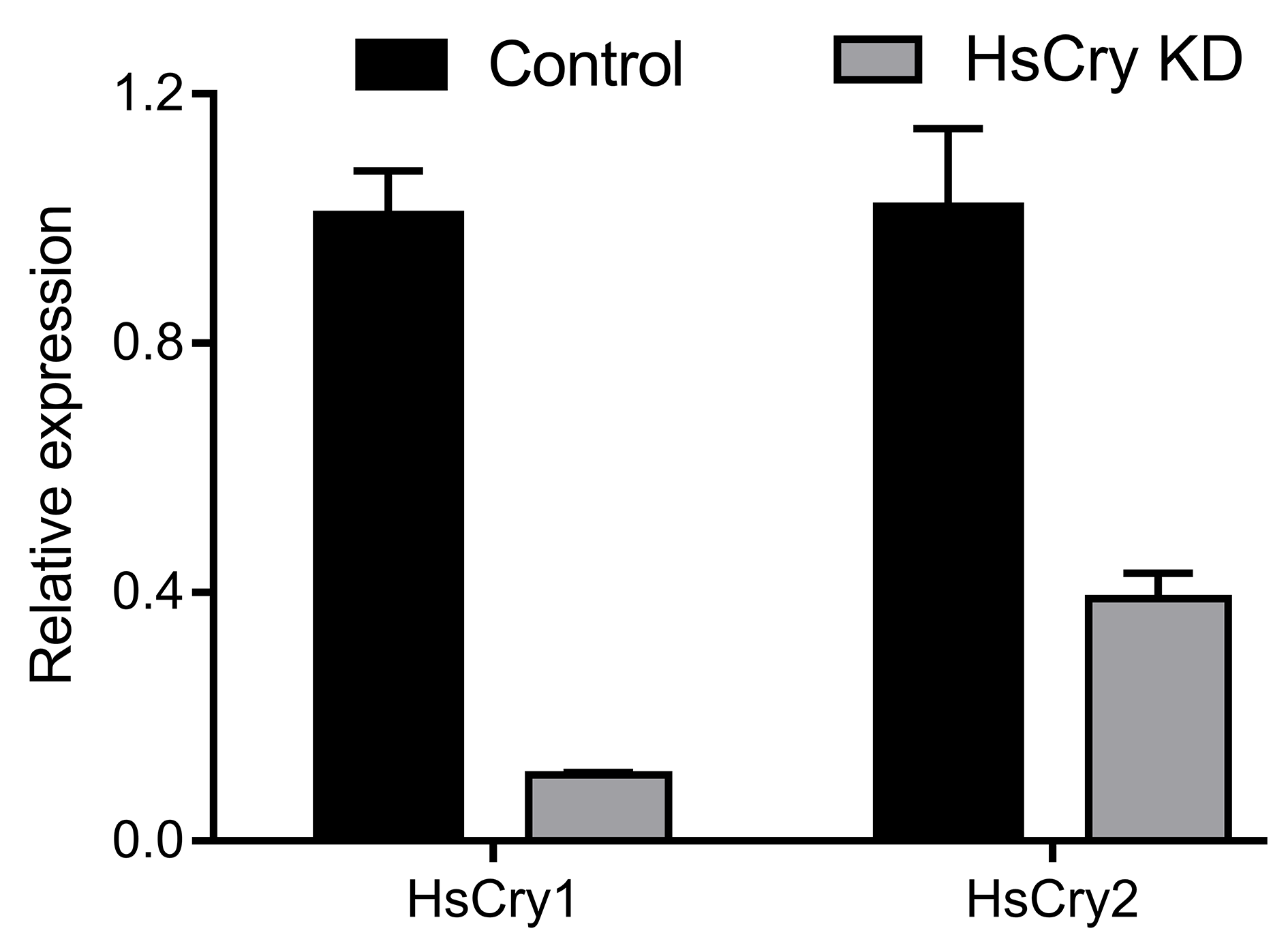

Supplement: S5 Fig — HSCRY1 and HSCRY2 gene expression is shown in control cells harboring the psiRNA-DUO-GFPzeo plasmid without shRNA insert (black bars) and compared to shRNA lines containing antisense constructs to HSCRY1 and HSCRY2 genes (HsCry KD: grey bars) constructed as described above. Primers used for qPCR analysis were as follows: HsCry1 Forward: 5’-GTGTTTCCCAGGCTTTTCAA-3’; HsCry1 Reverse: 5’-TGGTTCCATTTTGCTGATGA-3’; HsCry2F: 5-CTCGGAACAGTGCCTCAAATC-3; HsCry2 R: 5-GATAACGACCCTTCCACACAA-3. Data used to create graphs are in S2 Data. (TIF) [file pbio.2006229.s005.tif]

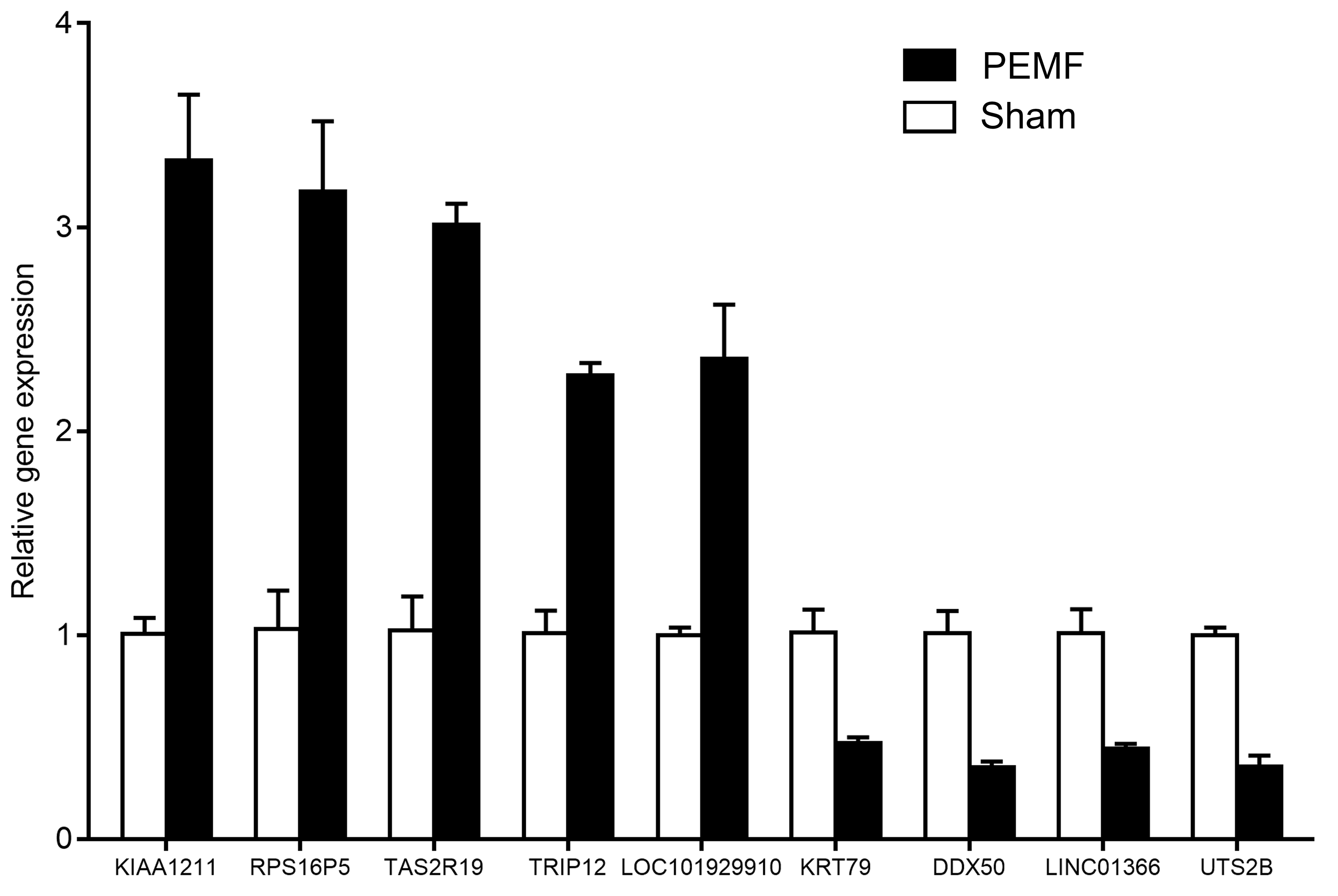

Supplement: S6 Fig — PEMF-treated (black bars) are compared with sham-treated (white bars) cells. Primers used and designation of accession numbers are described in S1 Table. Underlying data are in S2 Data. (TIF) [file pbio.2006229.s006.tif]

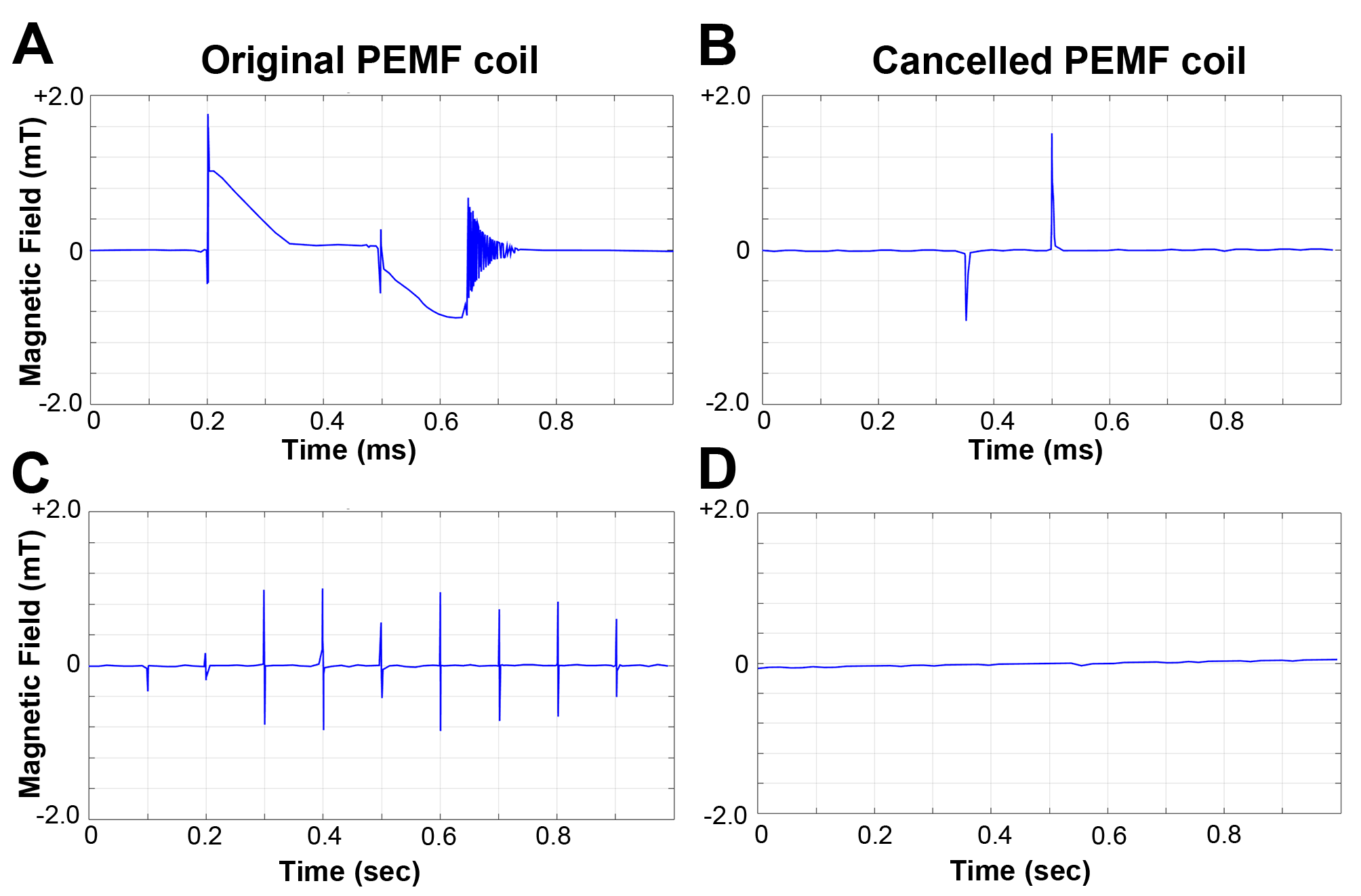

Supplement: S7 Fig — Panels A and B: magnetic field output is on a millisecond (ms) time scale. Shape of signal in the original coil (panel A) is compared to that in the cancelled PEMF coil (panel B). Note the exceedingly short (less than 0.01 ms) duration of the spike in the cancelled field condition compared to signal of the original coil (0.5 msec duration). Panels C and D are on a slower (second) time scale. Panel C represents the zoom out of signal in panel A from the PEMF coil. Panel D represents the zoom out of the signal from the cancelled coil in panel B. The signal is too short to be detected in the cancelled coil at this time scale. (TIF) [file pbio.2006229.s007.tif]

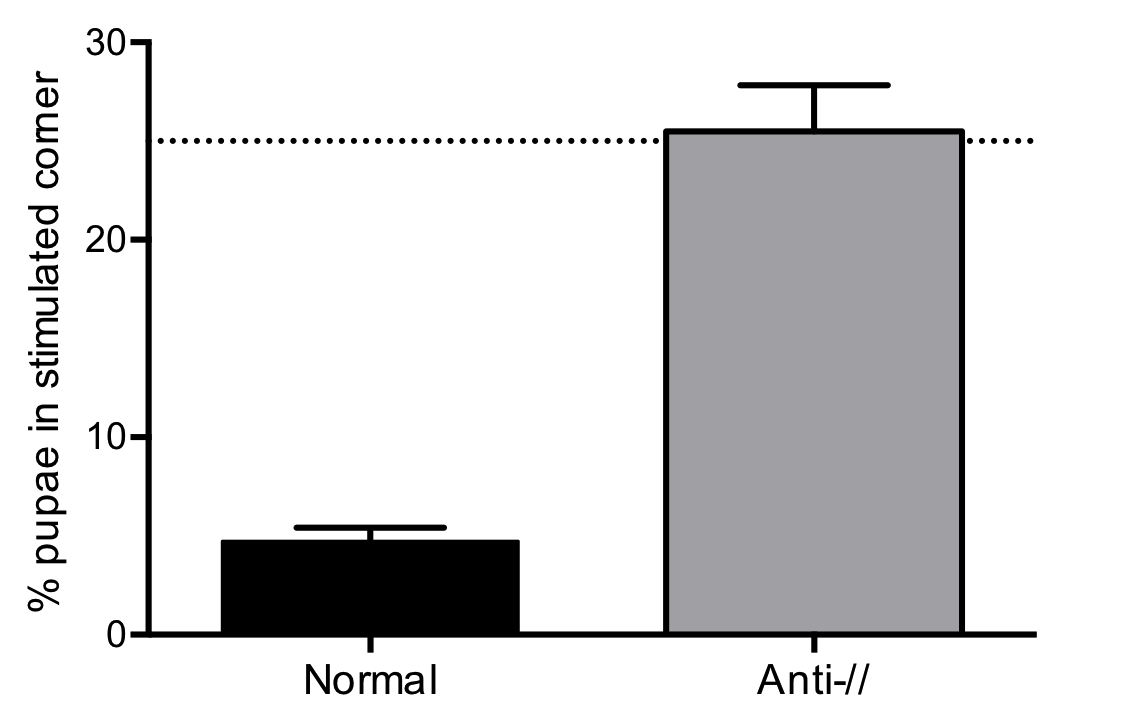

Supplement: S8 Fig — The percentage of pupae shown is that in the exposed petri plate corners as a percentage of pupae in all corners (see Materials and methods for full description of experimental procedure and analysis). The flies showed avoidance of corners exposed to pulsed magnetic field signal (see S1 Fig) but did not show avoidance to a cancelled PEMF signal using an antiparallel coil with cancelled magnetic field (see S7 Fig). The horizontal dotted line is 25%, i.e., the percentage of pupae present by chance, and the percentage in the PEMF corner is significantly reduced (MWU, p = 0.028). n = 4 independent biological experiments; error bar is SEM. Underlying data are in S2 Data. (TIF) [file pbio.2006229.s008.tif]

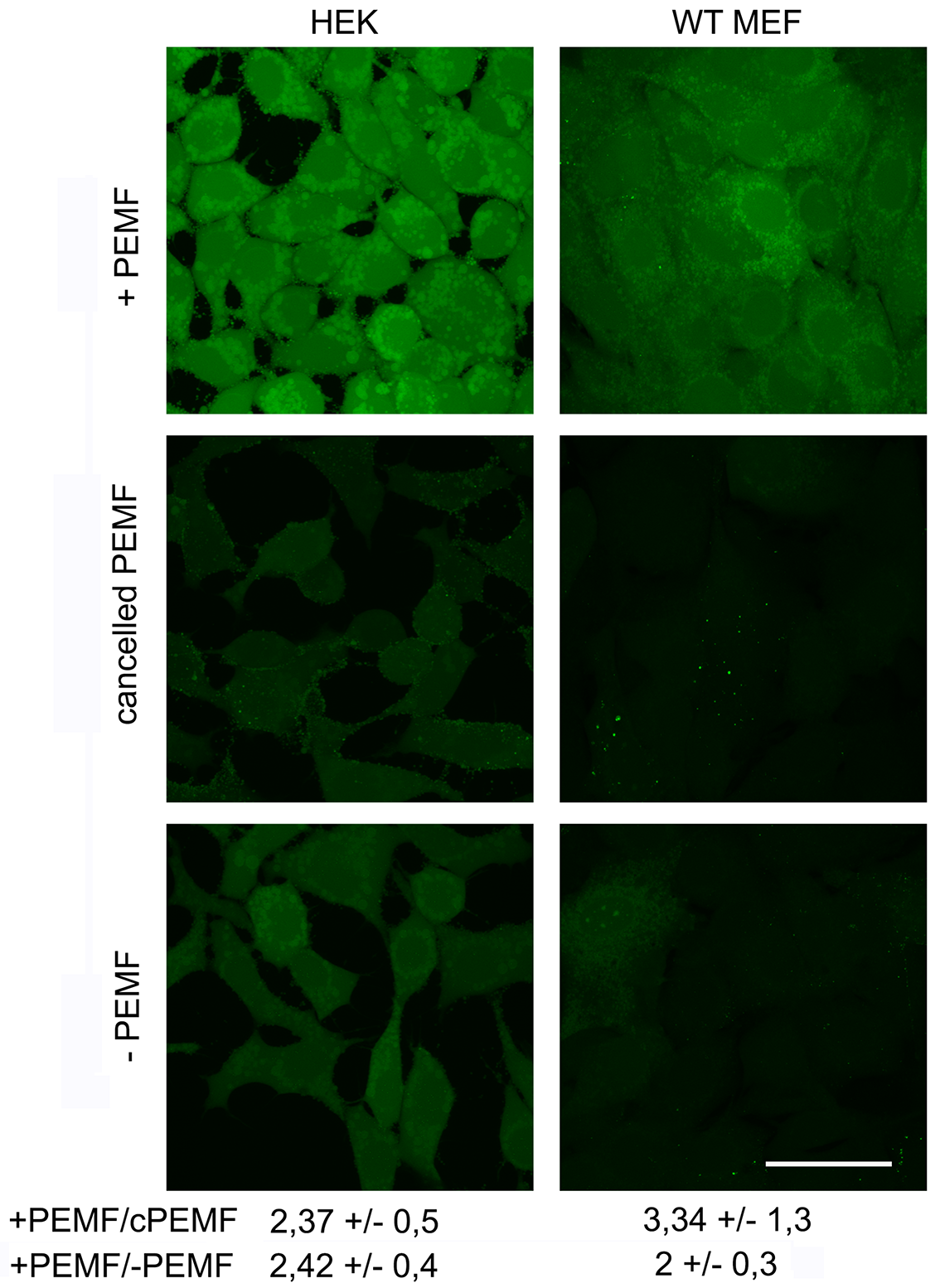

Supplement: S9 Fig — Living HEK293 or MEF were exposed either to PEMF (+PEMF) or cancelled PEMF (see S7 Fig for signal) for 15 minutes in darkness, simultaneously treated with DCFH-DA, then viewed by an inverted Leica TCS SP5 microscope. Control cell cultures (−PEMF) were treated in an identical manner but not exposed to either parallel or antiparallel PEMF coils (no exposure to any electrical or magnetic field). Images show a projection of all confocal z section. Scale bar 40 μm. Quantification of PMF effect is indicated by MFI ratio for each cell line (see Materials and methods). n = 5 independent biological repeats for all conditions. DCFH-DA, {5-(and-6)-chloromethyl-2’,7’-dichlorofluorecein diacetate}. (TIF) [file pbio.2006229.s009.tif]
